# Clinical Characteristics of Primary Repair for Perforated Peptic Ulcer: 10-Year Experience in a Single Center

**DOI:** 10.3390/jcm10081790

**Published:** 2021-04-20

**Authors:** Yun-Suk Choi, Yoon-Seok Heo, Jin-Wook Yi

**Affiliations:** Department of Surgery, Inha University Hospital, College of Medicine, Jung-gu, Inchon 22332, Korea; yunsukki@gmail.com (Y.-S.C.); gshur@inha.ac.kr (Y.-S.H.)

**Keywords:** PPU, primary repair, liver cirrhosis, Clavien-Dindo classification

## Abstract

Background: Perforated peptic ulcer (PPU) is a disease whose incidence is decreasing. However, PPU still requires emergency surgery. The aim of this study was to review the clinical characteristics of patients who received primary repair for PPU and identify the predisposing factors associated with severe complications. Method: From January 2011 to December 2020, a total of 75 patients underwent primary repair for PPU in our hospital. We reviewed the patients’ data, including general characteristics and perioperative complications. Surgical complications were evaluated using the Clavien-Dindo Classification (CDC) system, with which we classified patients into the mild complication (CDC 0–III, *n* = 61) and severe complication (CDC IV–V, *n* = 14) groups. Result: Fifty patients had gastric perforation, and twenty-five patients had duodenal perforation. Among surgical complications, leakage or fistula were the most common (5/75, 6.7%), followed by wound problems (4/75, 5.3%). Of the medical complications, infection (9/75, 12%) and pulmonary disorder (7/75, 9.3%) were common. Eight patients died within thirty days after surgery (8/75, 10.7%). Liver cirrhosis was the most significant predisposing factor for severe complications (HR = 44.392, *p* = 0.003). Conclusion: PPU is still a surgically important disease that has significant mortality, above 10%. Liver cirrhosis is the most important underlying disease associated with severe complications.

## 1. Introduction

Perforated peptic ulcer (PPU) is a disease in which acid, bile or food material spills into the peritoneal cavity through a perforated ulcer on the stomach or duodenal wall. It leads to pan-peritonitis, which is associated with high mortality due to sepsis. The incidence of surgery due to PPU is reported to be approximately 3–10 per 100,000 [1,2,3]. The total incidence of PPU has decreased since anti-acid drugs, such as H2 blockers or proton pump inhibitors (PPIs), have been used and since *H. pylori* eradication became standard [4,5]. Some studies have suggested that selected PPU patients can be treated by conservative therapy [6,7]. However, PPU is basically a surgical disease with serious morbidity and mortality that shows 30-day mortality up to 20% and 90-day mortality up to 30% [8,9,10,11,12].

The standard treatment of PPU consists of primary suture (associated or not with omentoplasty), without any type of vagotomy. Truncal vagotomy or highly selective vagotomy has been selectively considered to prevent recurrence of PPU. Only primary repair cannot guarantee long-term clinical course of PPU recurrence [8,13,14]. However, due to advancements in antiacid medication, primary repair without vagotomy is considered a suitable surgical option, especially for general surgeons who lack surgical skill to perform vagotomy. Laparoscopic surgery has been widely adopted in various surgical fields, including for the primary repair of PPU. It was first reported in 1990, and some studies have reported that laparoscopic primary repair for PPU showed rapid recovery without a difference in postoperative complications [15,16,17].

In this study, we summarize our 10-year experience with primary repair for PPU patients. To identify the clinical and laboratory findings associated with postoperative morbidity and mortality, we group our patients according to the Clavien-Dindo classification system [18]. We also evaluate the results of the laparoscopic surgery.

## 2. Materials and Methods

### 2.1. Data Collection and Patients’ Grouping

We reviewed medical data for PPU patients who underwent primary repair surgery at Inha University Hospital from January 2011 to December 2020. A total of 75 patients were enrolled for analysis, all of whom received emergency surgery within 24 h after hospital admission. We collected clinical data, including patients’ general characteristics (age, sex, body mass index (BMI, kg/m^2^)), personal history (smoking, alcohol abuse, NSAID use, steroid use), underlying disease, operation-related variables (surgery time, ulcer location, American Society of Anesthesiology (ASA) score), laboratory values (WBC, CRP, creatinine, hemoglobin, albumin, AST, ALT and glucose) from the operation day to postoperative days 1, 2, 3 and 5, and postoperative clinical course, including diet and surgical/medical complications. A positive alcohol history was defined as drinking more than 20 g of alcohol per week. Complications were evaluated by the Clavien-Dindo classification (Grade 0 to V). Two surgeons (Y.S. Choi and J.W. Yi) evaluated the Clavien-Dindo classification according to the patient’s medical record. We made two groups according to the CDC classification score as follows: the mild complication group included CDC grades 0 to III, and the severe complication group included CDC grades IV and V.

### 2.2. Statistics and Ethics

Statistical analysis was performed using SPSS version 22.0 (SPSS Inc., Chicago, IL, USA). The chi-square test or Fisher’s exact test was used for cross-table analysis according to sample size. Unpaired t-tests were used to compare the means between two clinical groups. To find the clinical variables associated with severe complications, logistic regression with the backward selection method was applied. The ethics of this study were approved by the Institutional Review Board of Inha University Hospital (IRB number: INH 2021-01-010).

## 3. Results

The clinical characteristics of the 75 PPU patients are described in Table 1. Their mean age was 54.5 years, and men were more prevalent than women (58/75 (77.3%)). Open surgery was performed in 61 cases, and laparoscopic surgery was performed in 14 cases. The mean operation time was 109.3 ± 40.8 min. Sipping water started 6.0 ± 4.3 days later, and a soft diet was permitted after 7.2 ± 4.4 days. The average hospital stay was 12.8 ± 11.4 days. Smoking and alcohol history were found in 53.5% and 49.3%, respectively. Seven patients (9.3%) had liver cirrhosis, twenty-three patients (30.7%) had hypertension, and thirteen patients (17.3%) had diabetes. Surgical complications occurred in 10 patients: 5 cases of surgical site leakage or fistula and 4 cases of wound complications. Among them, 7 patients received surgical intervention to correct the surgical complications. Medical complications occurred in 28 patients: infection in 9, renal complications in 4, hepatic complications in 2, and pulmonary complications in 7. According to the Clavien-Dindo classification, 14 patients were included in the severe complication group. Mortality occurred in 8 patients.

**Figure 1:**
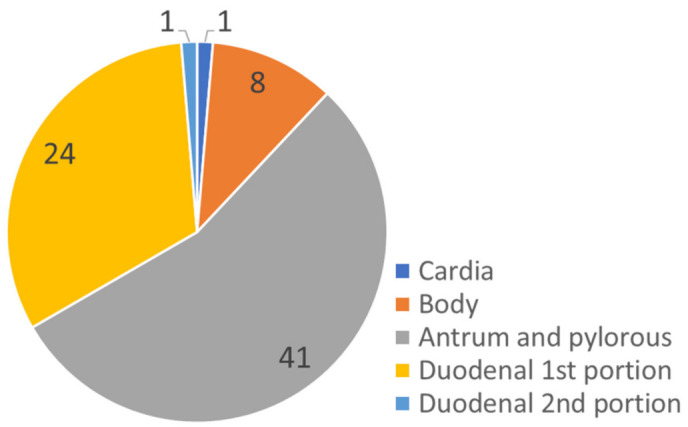
Anatomical location of the perforated peptic ulcer.

Fifty patients had gastric ulcer perforation, and twenty-five patients had duodenal ulcer perforation. The distribution of perforation sites is illustrated in Figure 1. Among the gastric ulcer patients, 41 patients had antrum and pyloric lesions, 8 had body lesions, and 1 had gastric cardia lesions. Duodenal ulcer perforation was mainly located on the first duodenal portion (24 cases), and only one case occurred in the second portion. Comparing the gastric and duodenal ulcer groups, alcohol use was significantly higher in the gastric ulcer group (29/50 (58.0%) versus 8/25 (32.0%), *p* = 0.034). Open surgery was preferred in the gastric ulcer perforation group (44/50 (88%) versus 17/25 (68%), *p* = 0.036). Other clinical variables were not significantly different between the gastric and duodenal ulcer groups.

We grouped our patients into mild complication (*n* = 61) and severe complication (*n* = 14) groups according to the Clavien-Dindo classification, as shown in Table 2. The severe complication group was older (65.43 ± 19.96 versus 51.97 ± 18.60, *p* = 0.019), had a higher proportion of women (7/14 (50%) versus 10/61 (16.4%), *p* = 0.007), and a higher ASA score (3.64 ± 0.63 versus 2.70 ± 0.67, *p* = 0.001). The proportions of smoking and alcohol use were significantly higher in the mild complication group. However, hypertension and liver cirrhosis were significantly more common in the severe complication group, as shown in Table 2. The surgical complication rate was higher in the severe complication group. Surgical site leakage or fistula occurred in five patients, and these patients required intensive care unit management. Six patients needed surgical intervention to correct complications. Medical complications in the severe complication group were significantly more often pulmonary, renal, hepatic, or infective.

Table 3 shows the clinical and underlying factors associated with severe complications, according to logistic regression. In the univariable analysis, open surgery, operation time, non-alcoholic history, liver cirrhosis, and hypertension were associated with severe complications. After multivariable model selection, liver cirrhosis was the only significant predictor for severe complications (odds ratio 44.392 (range 3.552–554.759), *p* = 0.003).

**Figure 2:**
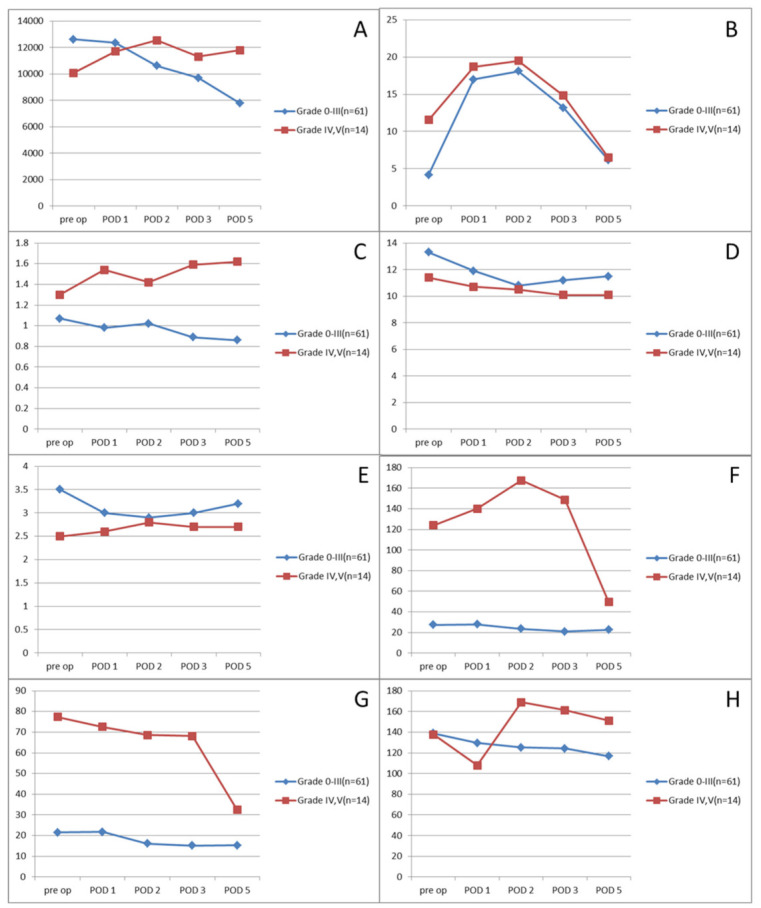
Selective laboratory findings according to the Clavien-Dindo classification. (**A**) WBC, (**B**) CRP, (**C**) creatinine, (**D**) hemoglobin, (**E**) albumin, (**F**) AST, (**G**) ALT, (**H**) glucose.

Preoperative and postoperative laboratory findings from the first to fifth days are described in Table 4 and illustrated in Figure 2. Preoperatively, the levels of CRP, AST, and ALT were significantly higher in the severe complication group, and the levels of hemoglobin and albumin were significantly lower in the severe complication group. During the follow-up, albumin was always significantly lower in the severe complication group, whereas AST and ALT were significantly higher in the severe complication group but normalized on postoperative day 5. The WBC count was not different preoperatively but returned to the normal range on postoperative day 5 in the mild complication group, at which time, it was significantly different from that in the severe group (7773.2 ± 3680.9 vs. 11,793.0 ± 3996.0; *p* = 0.004). CRP was significantly higher preoperatively in the severe complication group, but it decreased throughout the postoperative time, without showing a significant difference between the two groups. Creatinine levels were significantly higher in the severe complication group and worsened during the postoperative time, with significantly higher levels on postoperative days 3 and 5. The hemoglobin level was consistently higher in the mild complication group before and after surgery. Glucose did not show a significant difference between the two groups. However, the glucose level was consistently maintained in the mild complication group during the postoperative time, whereas it increased in the slope in the severe complication group.

Table 5 shows the clinical differences according to surgical approach, open (*n* = 61) versus laparoscopic (*n* = 14). Age (58.05 ± 18.16 versus 38.93 ± 17.67; *p* = 0.001) and ASA score (3.0 ± 0.7 versus 2.4 ± 0.9; *p* = 0.013) were significantly lower in the laparoscopic group. In the recovery course, sipping water (3.9 ± 1.6 days versus 6.5 ± 4.5 days; *p* = 0.001) and a soft food diet started significantly earlier (5.0 ± 1.5 days versus 7.7 ± 4.7 days; *p* = 0.001) in the laparoscopic group. Total hospital stay was also shorter in the laparoscopic group. Other variables did not show significant differences.

## 4. Discussion

We reported our 10-year experience with primary repair of PPUs in a tertiary medical center in Incheon, Korea. Most PPU patients are admitted to the emergency department due to severe abdominal pain. Recently, in Korea, the most common diagnostic modality has been abdominal computed tomography (CT). In our study, the gastric ulcer perforation rate was 67%, which was higher than the 43.6% to 52.0% of earlier studies [19,20]. The stomach body perforation rate (16%) was higher than that in a previous study (approximately 6.4%) in Korea but lower than that in Western populations [19,21]. Given the relatively high incidence of gastric ulcer perforation in the stomach body, we suggest that surgeons should focus on not only the antrum and duodenum, but also the stomach body to find perforation sites on CT scans before they perform PPU operations. Gastric ulcer perforation also frequently occurred in the patients with an alcohol use history in our analysis. Surgeons must also be aware of this factor when they make a differential diagnosis of PPU.

Surgical complications after primary repair were reported in approximately 13.3% of patients, with 6.7% having severe complications. A previous study reported that the surgical complication rate after primary repair for PPU ranged from 9.1% to 17%, similar to our results [17,22]. Furthermore, surgical results according to ulcer site, gastric vs. duodenal, were not significantly different after primary repair. According to these findings, primary repair is an acceptable operation method for PPU patients with either gastric or duodenal ulcer perforation. Gastric resection should be considered when the primary repair is impossible or unfavorable results are predicted, and if cancer perforation is highly suspected or confirmed by frozen biopsy, therapeutic gastrectomy should be performed.

We first dichotomized the surgical complications after primary repair, according to the Clavien-Dindo classification, as mild complications (0–III) and severe complications (IV, V). Factors associated with severe complications were older age, female sex, hypertension, and liver cirrhosis, as shown in Table 2. Liver cirrhosis was also an independent predictor (OR = 44.392) of severe complications in multivariable logistic regression, as detailed in Table 3. Some studies have shown that liver cirrhosis is an important risk factor for worse surgical outcomes after abdominal surgery [23,24,25,26,27]. Among our patients, all five patients with leakage or fistula had liver cirrhosis, and they received surgical intervention. In addition, among the eight patients who died, four patients had liver cirrhosis and death caused by septic shock. Therefore, patients with liver cirrhosis should be carefully monitored during surgery and postsurgical recovery.

Our laboratory findings also support the importance of liver function after surgery. Liver enzymes, such as AST and ALT, were significantly elevated in the severe complication group but recovered 5 days after surgery. Additionally, the albumin level was significantly lower in the severe complication group pre- and post-operatively. Altogether, the high OR of liver cirrhosis in logistic regression indicated that altered liver function was the most important factor predicting PPU patient morbidity and mortality according to our analysis. Other laboratory findings, such as WBC or CRP, were significantly normalized within 5 days postoperatively in the mild complication group. This finding suggests that recovery from PPU surgery takes approximately 5 days.

Currently, there is no consensus about the postoperative diet starting time. In our study, many surgeons performed primary repair for PPUs, and postoperative management was variable between surgeons. According to our findings that the inflammatory markers WBC and CRP were normalized within 5 days, it is worth considering diet initiation within 5 days after surgery. Furthermore, when surgery is performed by the laparoscopic approach, an earlier diet may yield better results than open surgery, as described in Table 5. Further study is required to establish the proper diet start time.

The surgical results of laparoscopic primary repair were comparable to those of open primary repair for PPUs. Laparoscopic surgery was associated with faster diet initiation, shorter hospital stays, and less surgical scarring (data not shown) in our analysis. Previous studies also described the advantages of laparoscopic primary repair of the PPU, such as in the wound complications, hospital stay, and postoperative pain [15,16,17,22,28,29,30]. Considering these benefits of laparoscopic surgery, it should be considered a preferred alternative for selected PPU patients.

Primary repair has the advantage of being easy to perform, but its weakness is the risk of PPU recurrence because vagotomy is not included, so it does not affect gastric acid secretion. In this study, there was a history of previous primary repair of PPU in 2 patients, and 1 patient underwent subtotal gastrectomy due to PPU recurrence 5 months after surgery. Although the probability of reoperation due to PPU recurrence is not high, follow-up and treatment, such as *H. pylori* eradication and antiacid medication, will be required.

This study had several limitations. This was a retrospective review analysis with a small number of patients. Comparisons between the variables may have limited statistical significance. The surgeries were performed by various general surgeons, and other surgeon factors differed, inconsistencies which should be eliminated in future studies. In the same context, postoperative management will vary between surgeons. However, due to the small sample size, adjusting the surgeon factor was impossible here. Future research with a large number of prospective analyses will be performed.

In summary, our study is the first to evaluate the surgical complications after primary PPU repair using the standardized Clavien-Dindo classification. Liver cirrhosis is an important predictor for severe complications. Finally, laparoscopic primary repair can show good surgical outcomes with early recovery in selected patients.

## Figures and Tables

**Table 1:** Clinical characteristics of patients.

Variable	Total (*n* = 75)	Gastric Ulcer (*n* = 50)	Duodenal Ulcer (*n* = 25)	*p*-Value
Age (years, mean ± sd)	54.5 ± 19.5	50.5 ± 18.9	56.5 ± 20.8	0.533
Sex				0.845
Male	58 (77.3%)	39 (78.0%)	19 (76.0%)	
Female	17 (22.7%)	11 (22.0%)	6 (24.0%)	
Body mass index (kg/m^2^)	21.9 ± 3.9	21.6 ± 3.6	22.4 ± 4.6	0.415
Approach method				
Open	61 (81.3%)	44 (88.0%)	17 (68.0%)	0.036
Laparoscopic	14 (18.7%)	6 (12.0%)	8 (32.0%)	
Operation time (minutes, mean ± sd)	109.3 ± 40.8	110.7±42.1	106.4 ± 38.8	0.67
ASA score ^*^	2.9 ± 0.8	2.9 ± 0.7	2.8 ± 0.9	0.332
Sipping water start time (postoperative days, mean ± sd)	6.0 ± 4.3	6.6 ± 4.9	4.9 ± 2.2	0.125
Soft food start time (postoperative days, mean ± sd)	7.2 ± 4.4	7.8 ± 5.1	6.0 ± 2.2	0.104
Hospital stay (total days, mean ± sd)	12.8 ± 11.4	12.0 ± 7.2	14.5 ± 17.0	0.388
Underlying condition				
Smoking	40/75 (53.3%)	30/50 (60.0%)	10/25 (40.0%)	0.102
Alcohol	37/75 (49.3%)	29/50 (58.0%)	8/25 (32.0%)	0.034
Liver disease	7/75 (9.3%)	4/50 (8.0%)	3/25 (12.0%)	0.575
Hypertension	23/52 (30.7%)	15/50 (30.0%)	8/25 (32.0%)	0.859
Diabetes	13/75 (17.3%)	6/50 (12.0%)	7/25 (28.0%)	0.084
Renal disease	1/75 (1.3%)	1/50 (2.0%)	0/25 (0.0%)	0.477
Pulmonary disease	5/75 (6.7%)	2/50 (4.0%)	3/25 (12.0%)	0.19
Cardiac disease	5/75 (6.7%)	2/50 (4.0%)	3/22 (12.0%)	0.19
Cerebrovascular attack history	6/75 (8.0%)	5/50 (10.0%)	1/25 (4.0%)	0.367
Cancer History	3/75 (4.0%)	1/50 (2.0%)	2/25 (8.0%)	0.211
Surgical Complication	10/75 (13.3%)	6/50 (12.0%)	4/25 (16.0%)	0.631
Leak or fistula	5/75 (6.7%)	3/50 (6.0%)	2/25 (8.0%)	0.743
Wound complication	4/75 (5.3%)	2/50 (4.0%)	2/23 (8.0%)	0.467
Require surgical intervention	7/75 (9.3%)	4/50 (8.0%)	3/25 (12.0%)	0.575
Medical Complication	28/47 (37.3%)	20/50 (40.0%)	8/25 (32.0%)	0.5
Pulmonary complication	7/75 (9.3%)	4/50 (8.0%)	3/25 (12.00%)	0.575
Renal complication	4/75 (5.3%)	3/50 (6.0%)	1/25 (4.0%)	0.716
Hepatic complication	2/73 (2.7%)	1/50 (2.0%)	1/25 (4.0%)	0.612
Infection complication	9/75 (12.0%)	6/50 (12.0%)	3/25 (12.0%)	1
Clavien-Dindo Classification				0.779
Grade 0	47/75 (62.7%)	31/50 (62.0%)	16/25 (64.0%)	
Grade I	2/75 (2.7%)	1/50 (2.0%)	1/25 (4.0%)	
Grade II	11/75 (14.7%)	8/50 (16.0%)	3/25 (12.0%)	
Grade III	1/75 (1.3%)	1/50 (2.0%)	0/25 (0.0%)	
Grade IV	7/75 (9.3%)	4/50 (8.0%)	3/25 (12%)	
Grade V	7/75 (9.3%)	5/50 (10.0%)	2/25 (8.0%)	

* American Society of Anesthesiologists score system.

**Table 2:** Clinical characteristics according to surgical complication status.

Variable	Mild Complication (Clavien-Dindo 0-III, *n* = 61)	Severe Complication (Clavien-Dindo IV-V *n* = 14)	*p*-Value
Age (years, mean ± sd)	51.97 ± 18.60	65.43 ± 19.96	0.019
Sex			0.007
Male	51 (83.6%)	7 (50.0%)	
Female	10 (16.4%)	7 (50.0%)	
Body mass index (kg/m^2^)	22.09 ± 3.65	20.83 ± 4.98	0.28
Operation time (minutes, mean ± sd)	106.72 ± 38.32	120.36 ± 50.55	0.356
ASA score	2.70 ± 0.67	3.64 ± 0.63	0.001
Underlying condition			
Smoking	36/61 (59.0%)	4/14 (28.6%)	0.039
Alcohol	34/27 (55.7%)	3/11 (21.4%)	0.021
Liver disease	3/61 (4.9%)	4/10 (28.6%)	0.006
Hypertension	15/61 (24.6%)	8/14 (57.1%)	0.017
Diabetes	9/61 (14.8%)	4/14 (28.6%)	0.218
Renal disease	1/61 (1.6%)	0/14 (0.0%)	0.63
Pulmonary disease	3/61 (4.9%)	2/14 (14.3%)	0.205
Cardiac disease	3/61 (4.9%)	2/14 (14.3%)	0.205
Cerebrovascular attack history	5/61 (8.2%)	1/14 (7.1%)	0.896
Cancer History	2/61 (3.3%)	1/14 (7.1%)	0.506
Surgical Complication	4/61 (6.6%)	6/14 (42.9%)	<0.001
Leak or fistula	0/61 (0%)	5/14 (35.7%)	<0.001
Wound complication	3/61 (4.9%)	1/13 (7.1%)	0.738
Require surgical intervention	1/61 (1.6%)	6/14 (42.9%)	<0.001
Medical Complication	14/61 (23.0%)	14/14 (100%)	<0.001
Pulmonary complication	2/61 (3.3%)	5/14 (35.7%)	<0.001
Renal complication	0/61 (0%)	4/14 (28.6%)	<0.001
Hepatic complication	0/61 (0%)	2/14 (14.3%)	0.003
Infection complication	1/61 (1.6%)	8/14 (57.1%)	<0.001

**Table 3:** Factors that associated with severe complications * (Clavien-Dindo classification IV and V).

	Univariable		Multivariable	
	Odds Ratio (95% CI)	*p*-Value	Odds Ratio (95% CI)	*p*-Value
Age (years, mean ± sd)	0.947 (0.874–1.026)	0.183		
Sex (reference: male)	16.361 (0.776–344.839)	0.072	4.585 (1.002–20.984)	0.05
Body mass index (kg/m^2^)	0.926 (0.679–1.263)	0.627		
Approach method (reference: open)				
Laparoscopic	0.014 (0.000–0.964)	0.048		
Operation time (minutes, mean ± sd)	1.040 (1.004–1.078)	0.029		
Underlying condition				
Alcohol	0.005 (0.000–0.474)	0.023	0.112 (0.012–1.082)	0.058
Smoking	2.746 (0.158–47.643)	0.488		
Liver Cirrhosis	128.435 (2.890–5708.296)	0.012	44.392 (3.552–554.759)	0.003
Hypertension	13.368 (1.387–128.801)	0.025	3.937 (0.912–16.987)	0.066
Diabetes	2.282 (0.157–33.152)	0.546		
Renal disease	0.000 (0.000–0.000)	1		
Pulmonary disease	1.424 (0.026–77.843)	0.863		
Cardiac disease	4.641 (0.124–173.355)	0.406		
Cerebrovascular attack history	0.105 (0.001–11.176)	0.344		
Cancer History	0.017 (0.000–2.465)	0.108		

* Logistic regression model with backward selection.

**Table 4:** Laboratory findings pre- and postoperatively, according to complication group.

Variable		Preoperative	POD * 1	POD 2	POD 3	POD 5
WBC (/µL)	Mild	12616.9 ± 6225.2	12348.3 ± 4576.3	10617.1 ± 4395.2	9698.3 ± 4635.6	7773.2 ± 3680.9
	Severe	10066.4 ± 4939.0	11702.1 ± 6363.1	12529.2 ± 6361.6	11300.9 ± 5464.1	11793.0 ± 3996.0
	*p*-value	0.157	0.464	0.172	0.225	0.004
CRP (mg/dL)	Mild	4.18 ± 7.58	16.99 ± 10.90	18.08 ± 7.98	13.17 ± 7.92	6.19 ± 5.08
	Severe	11.54 ± 13.31	18.70 ± 9.12	19.49 ± 5.66	14.80 ± 7.01	6.51 ± 2.72
	*p*-value	0.012	0.874	0.399	0.615	0.437
Creatinine (mg/dL)	Mild	1.07 ± 0.78	0.98 ± 0.63	1.02 ± 0.79	0.89 ± 0.64	0.86 ± 0.58
	Severe	1.30 ± 0.63	1.54 ± 0.88	1.42 ± 1.04	1.59 ± 1.50	1.62 ± 1.47
	*p*-value	0.076	0.005	0.127	0.049	0.028
Hemoglobin (g/dL)	Mild	13.3 ± 2.2	11.9 ± 2.35	10.8 ± 1.5	11.2 ± 2.1	11.5 ± 2.6
	Severe	11.4 ± 2.3	10.7 ± 2.4	10.5 ± 1.9	10.1 ± 1.7	10.1 ± 1.7
	*p*-value	0.009	0.062	0.311	0.058	0.02
Albumin (g/dL)	Mild	3.5 ± 0.7	3.0 ± 0.5	2.9 ± 0.4	3.0 ± 0.4	3.2 ± 0.4
	Severe	2.5 ± 0.7	2.6 ± 0.4	2.8 ± 0.3	2.7 ± 0.4	2.7 ± 0.4
	*p*-value	<0.001	0.005	0.446	0.033	0.009
AST (IU/L)	Mild	27.4 ± 16.7	27.9 ± 15.6	23.6 ± 11.6	21.0 ± 12.4	22.7 ± 18.9
	Severe	123.9 ± 222.1	140.1 ± 250.1	167.4 ± 463.8	148.6 ± 399.9	49.4 ± 63.7
	*p*-value	0.001	0.001	0.113	0.01	0.099
ALT (IU/L)	Mild	21.5 ± 13.4	21.7 ± 12.3	16.1 ± 8.0	15.2 ± 7.9	15.3 ± 9.6
	Severe	77.4 ± 103.5	72.6 ± 91.8	68.6 ± 147.5	68.1 ± 156.7	32.4 ± 38.4
	*p*-value	0.005	0.001	0.006	0.011	0.144
Glucose (mg/dL)	Mild	138.9 ± 48.3	129.5 ± 45.1	125.2 ± 35.9	124.3 ± 39.1	116.8 ± 33.8
	Severe	137.6 ± 48.9	108.0 ± 46.4	168.8 ± 100.9	161.2 ± 66.7	151.0 ± 68.2
	*p*-value	0.536	0.054	0.575	0.029	0.064

* POD: Postoperative days.

**Table 5:** Clinical characteristics according to surgical approach.

Variable	Open (*n*=61)	Laparoscopic (*n*=14)	*p*-Value
Age (years, mean ± sd)	58.05 ± 18.16	38.93 ± 17.67	0.001
Sex			0.406
Male	46 (75.4%)	12 (85.7%)	
Female	15 (24.6%)	2 (14.3%)	
Body mass index (kg/m^2^)	21.9 ± 4.1	21.6 ± 3.1	0.817
Operation time (minutes, mean ± sd)	105.7±40.3	125.0 ± 41.0	0.057
ASA score	3.0 ± 0.7	2.4 ± 0.9	0.013
Sipping water start time (postoperative days, mean ± sd)	6.5 ± 4.5	3.9 ± 1.6	0.001
Soft food start time (postoperative days, mean ± sd)	7.7 ± 4.7	5.0 ± 1.5	0.001
Hospital stay (total days, mean ± sd)	13.9 ± 12.4	8.1 ± 2.6	0.002
Surgical complication	9/61 (14.8%)	1/14 (7.1%)	0.45
Leak or fistula	4/61 (6.6%)	1/14 (7.1%)	0.937
Wound complication	4/61 (6.6%)	0/14 (0.0%)	0.325
Require surgical intervention	6/61 (9.8%)	1/14 (7.1%)	0.755
Medical Complication	25/61 (41.0%)	3/14 (21.4%)	0.172
Pulmonary complication	6/61 (9.8%)	1/14 (7.1%)	0.755
Renal complication	4/61 (6.6%)	0/14 (0.0%)	0.325
Hepatic complication	1/61 (1.6%)	1/14 (7.1%)	0.249
Infection complication	8/61 (13.1%)	1/14 (7.1%)	0.535
Clavien-Dindo Classification			0.799
Grade 0	36/61 (59.0%)	11/14 (78.6%)	
Grade I	2/61 (3.3%)	0/14 (0.0%)	
Grade II	9/61 (14.8%)	2/14 (14.3%)	
Grade III	1/61 (1.6%)	0/14 (0.0%)	
Grade IV	7/61 (11.5%)	0/14 (0.0%)	
Grade V	6/61 (9.8%)	1/14 (7.1%)	

## Data Availability

No new data were created or analyzed in this study. Data sharing is not applicable to this article.
